# Bibliography

**Published:** 1902-02

**Authors:** 


### Bibliography.

Studies of the Internal Anatomy of the Face. By M. H. Cryer, M.D., D.D.S., Professor of Oral Surgery, Department of Dentistry of the University of Pennsylvania. The S. S. White Dental Manufacturing Company, Philadelphia, 1901.

With this altogether too modest title the author ushers this book into the professional reading world. In the reviewer's opinion a more pronounced title would have been a better description of the real work of investigation made so laboriously and long into the true anatomy of the jaws and contiguous parts of the human subject. It is certainly true that Dr. Cryer has opened up new avenues of investigation long since supposed to be closed, or, in other words, that the last had been said in regard to the anatomy of the head, and that nothing more could be learned in this direction. The contents of this book prove very conclusively that it is never safe to rely unreservedly upon authority, for with new methods of procedure must come original investigations, with results possibly not in harmony with the older thought. The author says of this labor, "This investigation completely overturned the writer's conception of what was meant and what ought to be meant by the term typical. There is, doubtless, a typical or typal form for each bone, but it is not often found in nature. If we were to photograph a thousand temporal bones, for example, and make a composite of the entire number, the composite would properly be accepted as figuring the typal temporal. . . . This, to the writer's view, is strong testimony that the typal bone is ideal."

Much of this work has been made familiar in dental organizations in this country, through various papers read by the author and profusely illustrated by the lantern, but beautiful as these have been recognized to be, they did not exceed in clearness of detail the illustrations in this book. These certainly have been brought out with a perfection rarely excelled. This applies equally to the entire pictorial exhibit, extending to one hundred and fifty-one illustrations.

After "Introductory" and "General Considerations," the author takes up the critical examination of the lower jaw and thoroughly explains its anatomy and the connection of the teeth with the cancellated tissue. Fig. 8 very beautifully exhibits "the nerves and vessels within the cribiform tubes as they pass to the roots of the teeth."

The "Maxilla, or Upper Jaw," is next considered, and the variations are fully described, especially those connected with the antra. These variations are of especial importance to the practising dentist, for, relying upon the general anatomical descriptions, he will frequently be led into serious error in attempting operations upon these cavities. This portion of the work has never been better done; in fact, so far as the reviewer is aware, has not even been attempted to an equal extent, although some efforts have been made in this direction.

There has been so much wild talk of certain teeth being doomed to extinction, and even some writers have calmly considered the possibility of the human race becoming edentulous through the loss successively of the third molar, followed by the lateral and eventually the entire series, that it is quite refreshing, as well as a statement of a truth, when the author says, "Many skulls of the Caucasian races have only rudimentary third molars; in some skulls the third molar is entirely lacking. This has been received by many writers as evidence that the third molar teeth are being lost entirely, and as an indication that men will eventually become more or less edentulous. The author is of the opinion that the teeth of man are as good and fully formed at the present time as they were three thousand years ago; for if ancient Egyptian skulls are carefully examined, the rudimentary condition or complete suppression of the third molar will be found quite as frequently as in skulls belonging to relatively the same class of people to-day."

The impaction of teeth is well described and perfectly illustrated. This impaction so frequently leads up to serious reflex lesions that the subject-matter might well have been extended into some description of the pathological relations connected therewith, but if that were not in harmony with the intentions of the author, a more complete method of diagnosticating, especially in connection with the third lower molar, would enable the inexperienced to more readily find these misplaced teeth.

The author does not regard this work as a finality, but simply the beginning of still more laborious investigations. If he continues in this direction through other portions of the human subject, it will necessitate a revision of the anatomical studies of the earlier days made in the dissecting-room.

Some portions of this work of the author have been incorporated in Gray and other anatomical books.

The publishers have spared no effort in making this book worthy the subject.

Anatomy, Descriptive and Surgical. By Henry Gray, F.R.S., Fellow of the Royal College of Surgery, etc. Edited by T. Pickering Pick, F.R.C.S., and Robert Howden, M.A., M.B., C.M. A Revised American from the fifteenth English Edition. With seven hundred and eighty Illustrations, many of which are new. Lea Brothers & Co., Philadelphia and New York, 1901.

A review of this work would hardly be attempted by any one at this late date and in its fifteenth edition. Gray has become classical in medicine, and any follower of the healing art who fails to possess a copy or, at least, to be familiar with it cannot be considered quite up to the high standard of his profession.

Each edition of this work seems, however, slightly better than the next preceding, notwithstanding that it would appear almost an impossibility to add materially to it.

The section on Embryology has been given careful revision in this edition and "its text rendered more intelligible by the introduction of some sixty additional illustrations after His, Kollmann, Duval, and others."

It would seem difficult to add to the splendid illustrations for which Gray is celebrated, but this is continually being accomplished; and as the rapid sale necessitates frequent revision, the series will be added to in each edition in the future, materially increasing the value of the text.

The only objection that can be made to these constant additions is that the book is growing unwieldly, and, as a text-book, becoming more and more difficult to handle. The present edition numbers twelve hundred and fifty-seven pages. This applies only in the character of a text-book, for as a book of reference size is not important; indeed, adds to its value to the seeker after detailed information.

It is almost unnecessary to add that this edition is fully equal to all those that have preceded it in the care taken by the American publishers in having it presented to the medical reading public in the most satisfactory manner.

